# The Urine in Disease

**Published:** 1884-06-20

**Authors:** 


### *THE URINE IN DISEASE.*

A most excellent chart arranged by L. Lewis, M. D., M. R. C. S., England, giving qualitative tests, etc., of urine in disease, a very useful and practical chart for the practitioner's office. It is sent out as a supplement to The Medical World, a very excellent and practical journal of Philadelphia. Terms of Medical World, with chart, $1.00 per annum.

DOCTORS AND PATIENTS.—A bill has passed in Congress protecting physicians in the matter of not disclosing the confidential communication of their patients.

